# Effects of harvest treatments on forest carbon pools in eastern North America: A meta‐analysis

**DOI:** 10.1002/eap.70050

**Published:** 2025-05-26

**Authors:** Alexandre Collin, Evelyne Thiffault, Stéphane Tremblay, Frédérik Doyon, Philippe Nolet

**Affiliations:** ^1^ Institut des Sciences de la Forêt tempérée (ISFORT), Université du Québec en Outaouais Québec Canada; ^2^ Département des sciences du bois et de la forêt, Centre de Recherche sur les Matériaux Renouvelables (CRMR) Université Laval Québec Canada; ^3^ Direction de la Recherche Forestière, Ministère des Ressources naturelles et des Forêts Québec Canada

**Keywords:** carbon dynamics, carbon pools, clearcutting, forest carbon, forest harvesting, meta‐analysis, partial cutting

## Abstract

Understanding carbon dynamics in managed forest ecosystems is increasingly crucial for formulating informed recommendations in the context of climate change. Silviculture significantly impacts forest carbon pools, though these effects can vary depending on the type of treatment applied. In recent decades, partial cuttings have been proposed as an alternative to more intensive treatments like clearcutting to mitigate negative impacts on forest function and enhance carbon sequestration. In this study, we conducted a meta‐analysis comparing the effects of clearcutting and partial cuttings across North America on six forest carbon pools: live trees, snags, understory vegetation, coarse woody debris, forest floor, and soil mineral horizons. The analysis was based on a database of 558 carbon observations from temperate and boreal forests in eastern North America. Our findings indicate a −30% difference in total carbon post‐harvesting, predominantly influenced by changes in the overstory carbon pool. Only the live tree carbon pool was significantly affected by cutting intensity, with clearcutting resulting in lower total carbon values (−78% relative to the reference) compared to partial cuttings (−45%). However, after 30–40 years, live tree carbon levels were similar between clearcutting and partial cuttings. The primary factor influencing differences in deadwood carbon pools was the time since treatment, while soil carbon pools showed minimal variation with no significant differences compared to unmanaged forests. This meta‐analysis suggests that using partial cuttings instead of clearcutting to mitigate the effects of forest management on carbon pools may be more complex than previously thought and will depend on site conditions and allowing sufficient time for the forest to recover. Further studies are needed to identify suitable forest stands for partial cuttings and evaluate tree selection strategies that optimize forest productivity and carbon sequestration.

## INTRODUCTION

By covering 31% (4.06 × 10^9^ ha) of the world's total land area (FAO, 2020), forests mitigate significant greenhouse gas emissions through carbon (C) sequestration, and forests are estimated to offset around 30% of global fuel emissions (Birdsey & Pan, 2015). Such potential is locally variable and can be influenced by forest management through silviculture. By regulating the stand development, that is, species composition and structure through time, and improving the stand productivity, silviculture influences C stocks and fluxes in the living biomass, the necromass, and the soil beneath them (Ryan et al., 2010). However, such effects can vary considerably depending on the silvicultural treatment (Ameray et al., 2021; Zhang et al., 2010), the soils, the stand type, and the management regime (Wang et al., 2013), with notable interaction effects when these factors are combined (Rötzer et al., 2010).

Even‐aged management is the most commonly used silvicultural regime in North America and Europe (Keenan & Kimmins, 1993; Oswalt & Smith, 2014; Statistics Canada, 2018). Under even‐aged silvicultural systems, the overstory is usually removed at the end of the cutting cycle in one clearcutting operation (Smith et al., 1997). However, many nuances may be applied with some specific retention measures (Beese et al., 2003), allowing the passage of biological legacies through the next stand. The prevalence of clearcutting has been largely based on economic considerations, driven by the desire for greater harvested volumes per treated area (Vehkamäki, 1996) and the ease of artificial regeneration with suitable tree species. From an ecological perspective, clearcutting may seem to emulate natural catastrophic disturbances, such as wildfires and catastrophic windstorms, which kill most of the stems. However, this analogy has been widely debated (Kuuluvainen & Grenfell, 2012; Long, 2009; Seymour et al., 2002; Stockdale et al., 2016), particularly regarding its impact on carbon sequestration (Ameray et al., 2021). Following a high‐severity disturbance, forest ecosystems often experience net C losses due to increased soil respiration (Amiro et al., 2010), and regenerating stands may take several decades to achieve net C gains, whether the disturbance is natural or human‐caused (Covington, 1981; Senez‐Gagnon et al., 2018). However, clearcutting significantly differs from natural disturbances as transfers to the forest products sector reduce the C in the necromass and then in the soil organic carbon (SOC) pools. Because of the potential negative impacts on biodiversity (Martin et al., 2020), the risk of accelerated soil nutrient losses (Bormann et al., 1968), and mostly because of the negative esthetical perception of the viewshed altered by clearcutting (Ribe, 2005), a widespread public disapproval of that practice has emerged, calling for alternative forest management practices (Schneider et al., 2021).

Uneven‐aged management regimes offer an alternative by maintaining a permanent forest cover through regularly scheduled partial cuts. This approach mitigates the negative impacts on forest functions and ecosystem services often observed after clearcutting (Nolet et al., 2018; Peura et al., 2018). Moreover, forests managed under uneven‐aged regimes tend to be less susceptible to natural disturbances (Hanewinkel et al., 2014; Mohr et al., 2024), making this an effective adaptation strategy for mitigating the impact of climate change (Ameray et al., 2021, 2023). However, some species may be more vulnerable in the competitive environment created by uneven‐aged management, especially when water availability is projected to be limited (Ameray et al., 2023; Lafond et al., 2014). More specifically regarding C sequestration, partial cuttings are expected to enhance C sequestration (Ameray et al., 2021; Johnson & Curtis, 2001; Zhou et al., 2013) and offer higher levels of C storage at the forest landscape level (Assmuth & Tahvonen, 2018). For instance, living biomass and SOC pools have been found to be higher under uneven‐aged management in France (Jonard et al., 2017). However, the opposite trend has also been observed in some specific cases (Nilsen & Strand, 2013).

Although numerous meta‐analyses have examined the impacts of harvesting on forest C stocks, most have focused on the soil C pools or concentrations without specific comparisons between partial and clearcutting (e.g., Achat et al., 2015; James & Harrison, 2016; Nave et al., 2010). These syntheses report relatively small (<10%) reductions in soil C throughout the soil profile after clearcutting, with the greatest loss occurring in the forest floor (Mayer et al., 2020). However, the degree of this consistent negative effect varies widely between individual studies due to factors like soil type, climate, harvest treatment, or time since treatment. Meta‐analyses focusing on other C pools are less common. Zhou et al. (2013) observed significant effects of partial cuttings on aboveground biomass dynamics (e.g., increased dbh and decreased aboveground C biomass) in various forest types worldwide. Still, they noted substantial variability due to climate zones and forest types. Although these studies concentrating on specific C pools are informative, they do not provide a comprehensive understanding of the entire ecosystem C dynamics and how these different pools change relative to one another over time. Kalies et al. (2016) conducted a more comprehensive meta‐analysis on the impact of various management practices (e.g., thinning, burning, harvesting) on multiple forest ecosystem C pools and found no significant differences between partial and clearcutting. However, only a few individual studies that reported partial harvests were included in the meta‐analysis, resulting in a high level of response ratio (RR) variability that may have obscured potential differences. Overall, there are still important knowledge gaps in our understanding of the impact of harvesting treatment, especially partial cuttings, on the dynamics of various forest C pools (Goetz et al., 2012; Mason et al., 2021).

While meta‐analyses are valuable instruments for synthesizing global responses, their amalgamation of diverse treatments and study designs from across the world in pursuit of broader generalizations may lead to a trade‐off, where finer distinctions, particularly those that are region specific, might be forfeited (James et al., 2021). Additionally, meta‐analysis is inherently cumulative and requires regular updates as more papers are published to reduce variability and enhance the robustness of the conclusions.

In this study, we conducted a meta‐analysis to specifically examine the response of various forest ecosystem C pools (i.e., live trees, snags [dead standing trees], understory vegetation, coarse woody debris, forest floor, and soil mineral horizons) following partial cuttings or clearcutting in eastern North American forests relative to uncut conditions. Our approach concentrated on a well‐defined region encompassing boreal and northern temperate ecosystems, allowing us to standardize treatment conditions and thus undertake a more comprehensive examination of the nuances between partial cuttings and clearcutting across different time points following treatment and between forest biomes.

## METHODS

We utilized the online Web of Science and Google Scholar databases to search for relevant scientific peer‐reviewed papers published by the end of 2023. Keywords used included a combination of “forest,” “carbon,” “cutting,” “harvest,” and “management” (in the title, abstract, or keywords). Papers were initially selected based on their geographic location, focus on C content or biomass, and the use of harvest treatments such as partial cuttings and/or clearcutting. Modeling studies were excluded to focus our meta‐analysis on observational data only. To ensure we did not overlook potential papers, we also examined the references cited in the papers selected from our initial search. Out of 669 screened articles, 89 were initially retained before a second screening.

**FIGURE 1:**
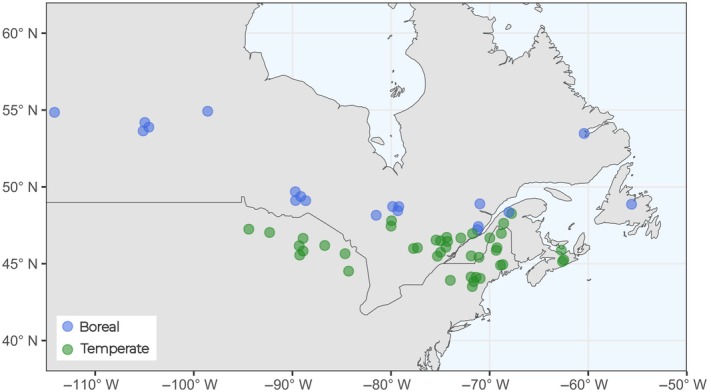
Distribution of the experimental sites used in this study. Green circles represent sites reported as being in temperate forests, while blue circles represent those in boreal forests. Circles are semitransparent such that darker colors indicate overlap of multiple studies.

We applied the following criteria to select data from these articles for our meta‐analysis. First, studies without uncut forest plots (i.e., controls) were excluded as they do not allow for the computation of a relative difference in cutting relative to local conditions over time. An exception was made for chronosequences without controls by using the last measure or the oldest stand as the control (>80‐year‐old stands). Second, studies that did not report C data as quantities/stocks per area, such as in megagrammes of carbon per hectare, or that could not be converted into this format were excluded to ensure the standardization of the observed response. The rationale was that some studies yielded different responses to the treatment depending on the measurement unit. For example, in their meta‐analysis, Nave et al. (2010) found different harvest effects on soil C when the studies expressed it as concentration instead of pool size. However, most studies focusing on tree dynamics after a cutting treatment were expressed in terms of biomass, volume, or tree diameter and density. These data were first manually converted to biomass values using diameter‐based allometric equations of Lambert et al. (2005) or volume‐based equations of the Canadian National Forest Inventory (Boudewyn et al., 2007). The C content was then obtained by multiplying the obtained biomass (in megagrammes per hectare) by 0.5, a commonly applied method for converting biomass into C content (Penman et al., 2003). We also excluded articles that used soil preparation and/or fertilization treatment after cutting, as we only wanted to infer the effect of harvest treatments alone. After the second screening, a total of 61 articles were identified as relevant with enough information to be included in a meta‐analysis. These publications correspond to forest harvests conducted in boreal and temperate forests of eastern North America. A list of basic information about each publication is available in Appendix S1: Table S1, while the geographic locations of the experimental plots reported in these publications are provided in Figure 1.

For each publication, the harvest treatments were classified as either clearcutting or partial cutting. However, it is important to note that “partial cutting” is a generic term encompassing a wide range of silvicultural treatments and intensities (Bose et al., 2013). In North America, this can refer to cutting with a focus on (1) natural seedling establishment to create an even‐aged (regular shelterwood) or uneven‐aged stand (irregular shelterwood, selection cutting) or (2) promoting growth of remaining trees in an even‐aged stand (commercial thinning). Among these, irregular shelterwood and selection cutting belong to continuous‐cover silviculture with the aim of retaining a permanently irregular structure (Mason et al., 2021). The term clearcutting can refer to slightly different methods depending on the intended outcome. It may involve removing all merchantable trees with little attention to regeneration (commercial clearcutting) or removing trees while protecting the soil and ensuring natural or planted regeneration (Windmuller‐Campione et al., 2020). Most studies in the literature have focused on commercial clearcutting or did not provide enough details to determine the specific method used. Therefore, we chose to retain the generic term clearcutting in this study. However, studies explicitly describing clearcutting combined with soil preparation and/or artificial regeneration were excluded from this meta‐analysis.

Multiple C values were recorded for each publication depending on the number of harvest treatments tested, time since treatment, and stocks sampled. This dataset represented a total of 558 C observations from 360 experimental units. To combine data across studies, an effect size was calculated as the log RR for each treatment from each study (Hedges et al., 1999; Lajeunesse, 2011). The effect size was calculated as follows:

(1) $$\mathrm{RR}=\ln\left(\frac{\bar{X_{T}}}{\bar{X_{C}}}\right)$$

where $\bar{X_{T}}$ is the mean C content at a specific time since treatment of an individual harvest treatment and $\bar{X_{C}}$ is the corresponding mean C content of the reference (old or unmanaged stand). In our study, “time since treatment” refers to the time elapsed since the most recent cutting. Therefore, when a study reported C pool values across multiple cutting cycles, we only considered the values from the last cycle for both the treatment and reference stands. The SE of the RR was calculated as follows:

(2) $$\mathrm{SE}\left[\mathrm{RR}\right]=\sqrt{\frac{\mathrm{SE}{\left[\bar{X_{T}}\right]}^{2}}{{\bar{X_{T}}}^{2}}+\frac{\mathrm{SE}{\left[\bar{X_{C}}\right]}^{2}}{{\bar{X_{C}}}^{2}}}$$

The effect size is a unitless measure; thus, it is often back‐transformed as a percentage difference relative to the reference to enhance the overall biological interpretation of the treatment effect. The percentage difference was calculated as follows:

(3) $$\text{percent difference}=\left(e^{\left(\mathrm{RR}\right)}-1\right)\times 100$$

After calculating the effect size for each C observation, the next step was to identify the categorical variables (i.e., C pool, cutting practice, time since treatment, forest biome, reference type) that best explained the variation in measured C. To achieve this, we applied a variance‐partitioning approach similar to an ANOVA, as recommended by Hedges and Olkin (1985). This involved calculating within‐group (*Q*_*w*_) and between‐group (*Q*_*b*_) heterogeneity for the RR values associated with each predictor. A categorical variable with a large and statistically significant *Q*_*b*_ was considered a strong predictor of variation in effect size.

To determine which variable had the greatest explanatory power, we first performed this analysis on the entire RR dataset, identifying the variable with the highest and most significant *Q*_*b*_. We then followed a hierarchical approach: the dataset was divided based on the strongest predictor, and the variance‐partitioning procedure was repeated within each subgroup. This iterative process continued until no additional categorical variables were found to be significant.

After identifying the best predictors, we analyzed the calculated RR between harvest treatments using mixed‐effects meta‐regression models (Sera et al., 2019). In these models, harvest treatment was considered the fixed effect, while biome and time since treatment were both considered as random effects. The models utilized the calculated RR and the associated uncertainty estimates (SE[RR]), which considered the variability and number of replicates from each study. The effects on total ecosystem C were also calculated, but only using 12 publications (46 observations) that measured C in at least live trees, soil, and coarse woody debris (pools the most contributive to total C).

Unfortunately, estimates of the SD and sample size for each $\bar{X_{T}}$ and $\bar{X_{C}}$, which are essential for computing SE[RR] and conducting the most robust meta‐analyses (i.e., weighted method), were available for only 81% of the total observations recorded in the retained publications. In order to retain as much available data as possible, we also conducted mixed‐effects models on an unweighted meta‐analysis (i.e., without SE[RR]) and compared the results with those of the weighted meta‐analysis. Using this unweighted method, we performed non‐parametric resampling techniques (bootstrapping) to compute CIs around the mean effect sizes to ensure robust estimation (Adams et al., 1997). This unweighted method was also used to evaluate the dynamics of mean effect sizes over time for each carbon pool. For pools that exhibited different relationships with time between harvest treatments, we confirmed these relationships by conducting a weighted meta‐regression on a subset of observations that provided the necessary information. Various models were tested, and we selected the significant ones with the lowest Akaike information criterion (AIC).

All statistical analyses were completed using R software version 4.2.3 (R Core Team, 2023). Mixed‐effects meta‐regression models were performed using the *rma* and *rma.mv* functions from the *metafor* package (Viechtbauer, 2010). Mixed‐effects models on unweighted meta‐analysis were performed using the *lme* function of the *nlme* package (Pinheiro, 2022), while bootstrapping was computed using the *bootstrap* function of the *lmeresampler* package (Loy et al., 2023).

## RESULTS

### Data overview and primary sources of carbon variation

The effect size values used for our meta‐analysis were relatively well‐proportioned between biomes. However, data were generally more available for clearcutting than for partial cuttings, particularly in boreal forests. Among the data recorded in the boreal forest (43.1% of the total), only 10.3% were under partial cuttings, whereas this percentage was twice as high in temperate forests. Such distribution reflects the standard silvicultural approach in the temperate forests, where partial cuttings are applied to maintain multi‐cohort stand structure. Data availability was also influenced by the C pool, with the majority of data pertaining to live trees (25.4%), forest floor (20.1%), and deadwood biomass (19.2% for coarse woody debris and 15.3% for snags). In comparison, data were scarce for the soil mineral horizons and the understory vegetation (11.4% and 8.7%, respectively). It is also noteworthy that reported C values for soil mineral horizons differed in the sampling depth, ranging from 7 to 150 cm, with a median sampling depth of 45 cm.

Regarding the time series, C values were available for a longer period after treatment in clearcutting stands compared to partial cutting ones. In clearcutting, the effect sizes were calculated based on a median of 15 years since treatment, with a maximum duration of 110 years. Approximately three‐quarters of the values were under 34 years. In partial cutting, the median time since treatment (9 years) was relatively similar to clearcutting, but approximately three‐quarters of the values were only under 12 years, with a maximum value of 50 years.

**FIGURE 2:**
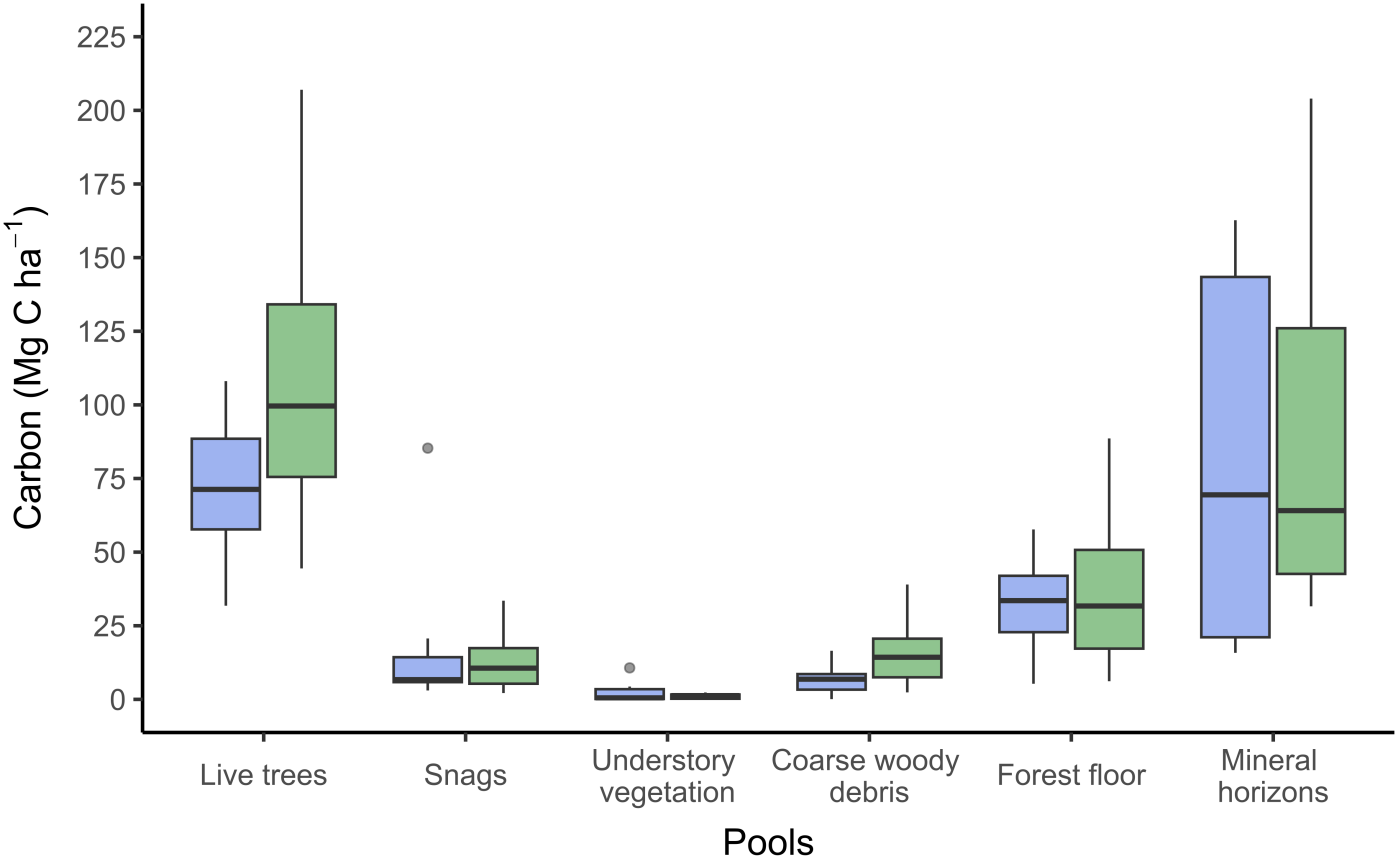
Carbon quantities by pool in temperate (green) and boreal (blue) forests reported among the reference plots of the experimental studies.

The total ecosystem C stock (i.e., the addition of all measured C pools in this study) in the forests used as a reference for the effect size calculations averaged a total of 223.9 Mg C ha^−1^ (Figure 2). Despite considerable variability, live trees and the soil mineral horizons contributed the most to the total C (40.9% and 33.6%, respectively) and were significantly higher than other pools (*p* < 0.001). The forest floor (14.24%) had the third‐highest pool values, followed by deadwood (5.76% for snags and 4.74% for coarse woody debris) and understory vegetation (0.72%).

Our meta‐analysis revealed several significant sources of C variation that differed among the pools considered in managed forests (Table 1). As anticipated from Figure 2, the C pool emerged as the most influential factor determining the variation in effect size, followed by the time elapsed since harvesting. When considering the pools independently, we observed that the variability in effect size for aboveground biomass (i.e., live trees and snags) was primarily influenced by the harvest treatment, even without accounting for the time since treatment. Effect size values for coarse woody debris were mainly predicted by the time since treatment, while forest floor C was solely affected by the biome. The effect size calculated for the soil mineral horizons did not exhibit any significant variability from any categorical variable considered in this study, including the sampling depth (*Q*_*b*_ = 0.29, *p* = 0.589).

**TABLE 1:** Between‐group heterogeneity (*Q*_*b*_) among the *k* studies for each response parameter.

Response parameter	*k*	C pool	Cutting treatment	Time	Biome
Ecosystem C	457	967.77*	1.17	7.66*	0.41
Live trees	103	…	338.74*	102.72*	51.88*
Snags	63	…	63.20*	13.17*	0.038
Understory vegetation	35	…	4.75*	6.46*	16.21*
Coarse woody debris	99	…	1.34	73.13*	55.81*
Forest floor	97	…	0.71	0.26	26.47*
Mineral horizons	60	…	0.046	1.16	0.45

* Significant effect (*p* < 0.05) of the categorical variable on the response parameter considered.

It is noteworthy that the forest stands used as references in each study, and for the effect size calculation, varied significantly in their characteristics. Approximately 55% of these reference stands were either old forests or areas that have no recorded evidence of past management activities. The remaining 45% were areas that had been cut more than 80 years ago and left unmanaged since then (Appendix S2: Table S1). This categorical variable introduced significant variability in all stocks except understory vegetation and the mineral horizons. However, this factor exhibited a high correlation with biome and practice type. Clearcutting stands predominantly utilized old forests as a reference stand. In contrast, partial cutting stands mainly used unmanaged forests as a reference in boreal forests, and a more balanced mix of old and unmanaged forests in temperate forests (Appendix S2: Table S1). Such intercorrelated variables complicate the data analysis because attempting to eliminate the effect of one random factor will inevitably remove some variability explained by more ecologically important factors, thus introducing bias into the results. However, variance partitioning of the data revealed that the explained variation in effect size values by the type of reference stand used was already fully accounted for by harvest treatment and biome type. When considered independently, the reference type used did not show significant explained variance (*p* = 0.866), so we chose not to include it in the subsequent analyses.

### Effect of harvest treatments by biome on each carbon pool

**FIGURE 3:**
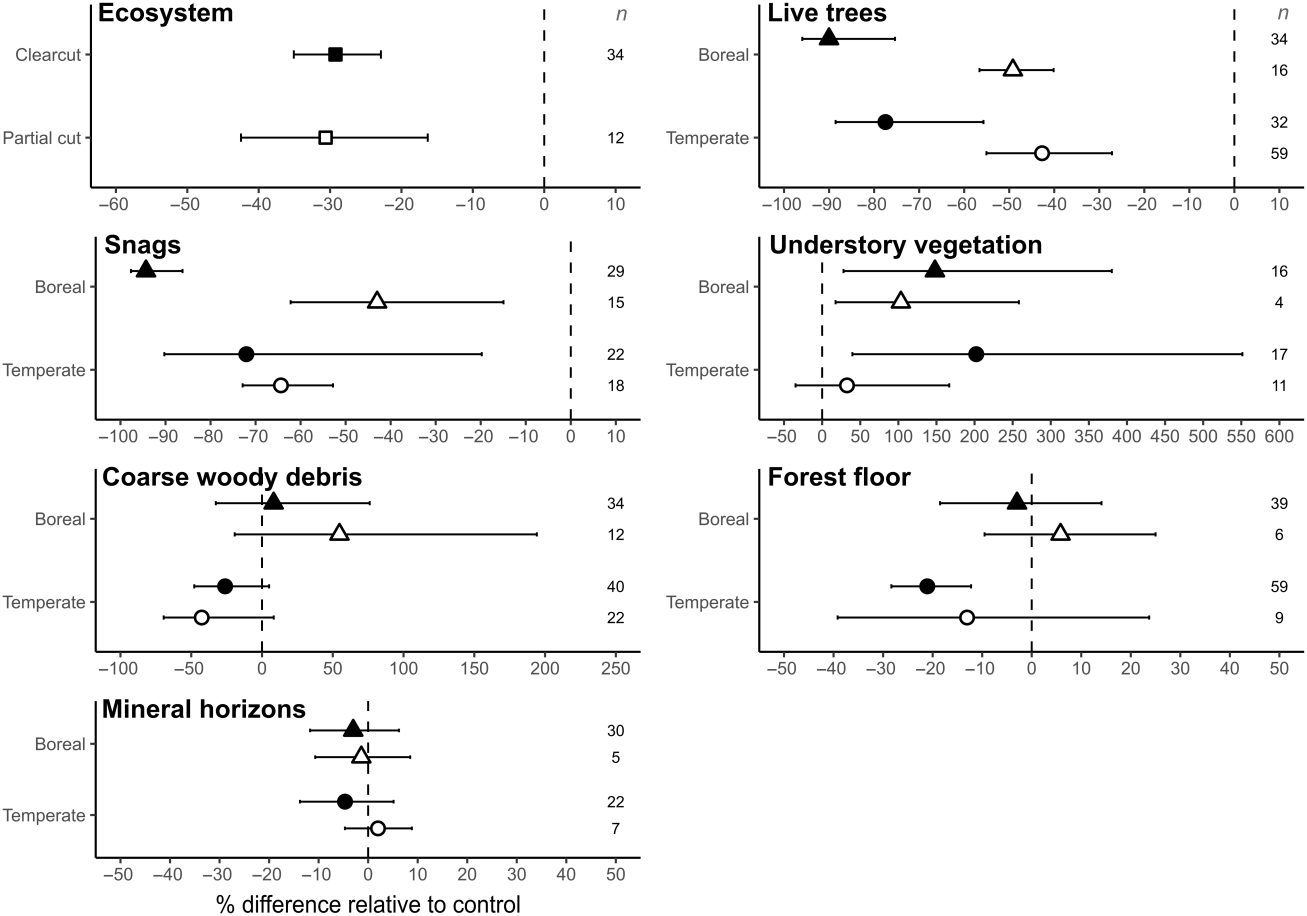
Effect of clearcutting (black symbols) and partial cuttings (white symbols) on the percentage difference of C relative to control between pools and forest biome (points: boreal, triangle: temperate). Each mean estimate is shown with a bootstrapped 95% CI. Each interval that does not overlap with the dotted vertical line indicates a statistically significant difference from the reference stand (i.e., old or unmanaged stand), while non‐overlapping intervals between treatments indicate significant differences between them. The number of observations from the literature included in each estimate is listed on the right of each panel. Time since harvest treatment was included as a random effect in the model.

Averaged across 12 publications that recorded enough C pools to compute an ecosystem component, forest harvesting resulted in a significant reduction in total ecosystem C compared with a reference (old or unmanaged stand) (−30%; Figure 3). However, no differences were found between clearcutting and partial cuttings. As anticipated from Table 1, only the aboveground stocks are affected by harvest treatments. When controlling for the time since treatment, C in live trees was still significantly affected by the harvest method, irrespective of the biome considered, with clearcutting resulting in a greater reduction (−78%) than partial cuttings (−45%) relative to the reference. The effect size on the C pool of snags showed a similar response, although high variability was observed in temperate forests. This variability was likely introduced by specific differences in each harvest method. For example, some publications studied clearcutting with the removal of snags, while others left them on‐site (clearcutting with snag retention), which obviously greatly influenced the C values in this pool (Appendix S2: Figure S1 for a specific analysis on a small subset of observations where this information was available). Except for partial cuttings in temperate forests, the understory vegetation experienced a significant increase in the C pool following harvesting, averaging 121%, with high variability mainly caused by the low amount of observations available. The main predictor of forest floor C pool was the biome type (refer to Table 1), and we observed a significant difference of −21%, but only in the clearcutting treatment of temperate forests (Figure 3). This effect in temperate forests still appeared even when harvest treatments were pooled and was mostly driven by the higher hardwood content (Appendix S2: Figure S2). The effect sizes on the C pool of coarse woody debris and mineral horizons did not exhibit any differences from the reference due to the harvest treatment or biome type. These results were computed using the entire available database, which constrained us to use unweighted meta‐analysis due to the lack of variance information. A weighted meta‐analysis, computed on 81% of the observations with the required information, yielded roughly the same results, and differences can be seen Appendix S2: Figure S3.

### Carbon pool dynamics following harvesting

**FIGURE 4:**
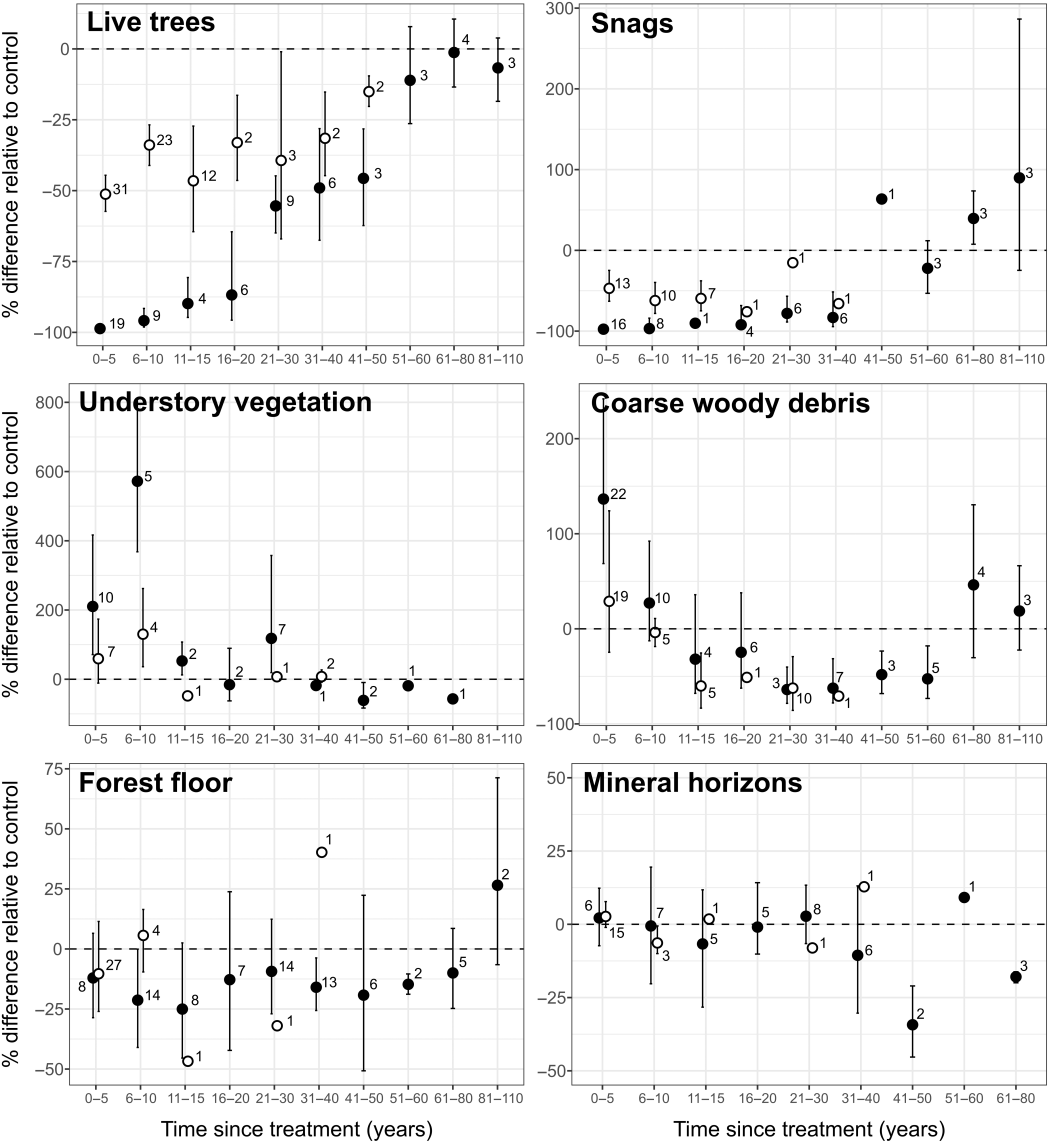
Effect of time after clearcutting (black circles) and partial cuttings (white circles) on the percentage difference of C relative to control between pools. Each estimate is shown with a bootstrapped 95% CI. Each interval that does not overlap with the dotted vertical line indicates a statistically significant difference from the reference stand (i.e., old or unmanaged stand), while non‐overlapping intervals between treatments indicate significant differences between them. The number of observations from the literature included in each estimate is listed next to the circles.

Time since treatment emerged as a significant descriptor of C pool variability observed in total aboveground as well as deadwood biomass (refer to Table 1, Figure 4). Live trees were the only C pool that showed differences between harvest practices over time. Values of percentage of change from partial cuttings treatments showed a steady increase from −50% to −25% over a 50‐year period. By contrast, clearcutting treatments began with a −100% reduction in C values compared to reference stands and then quickly increased to match partial cuttings levels after approximately 30 years since treatment before reaching reference levels at 60 years after harvest.

**FIGURE 5:**
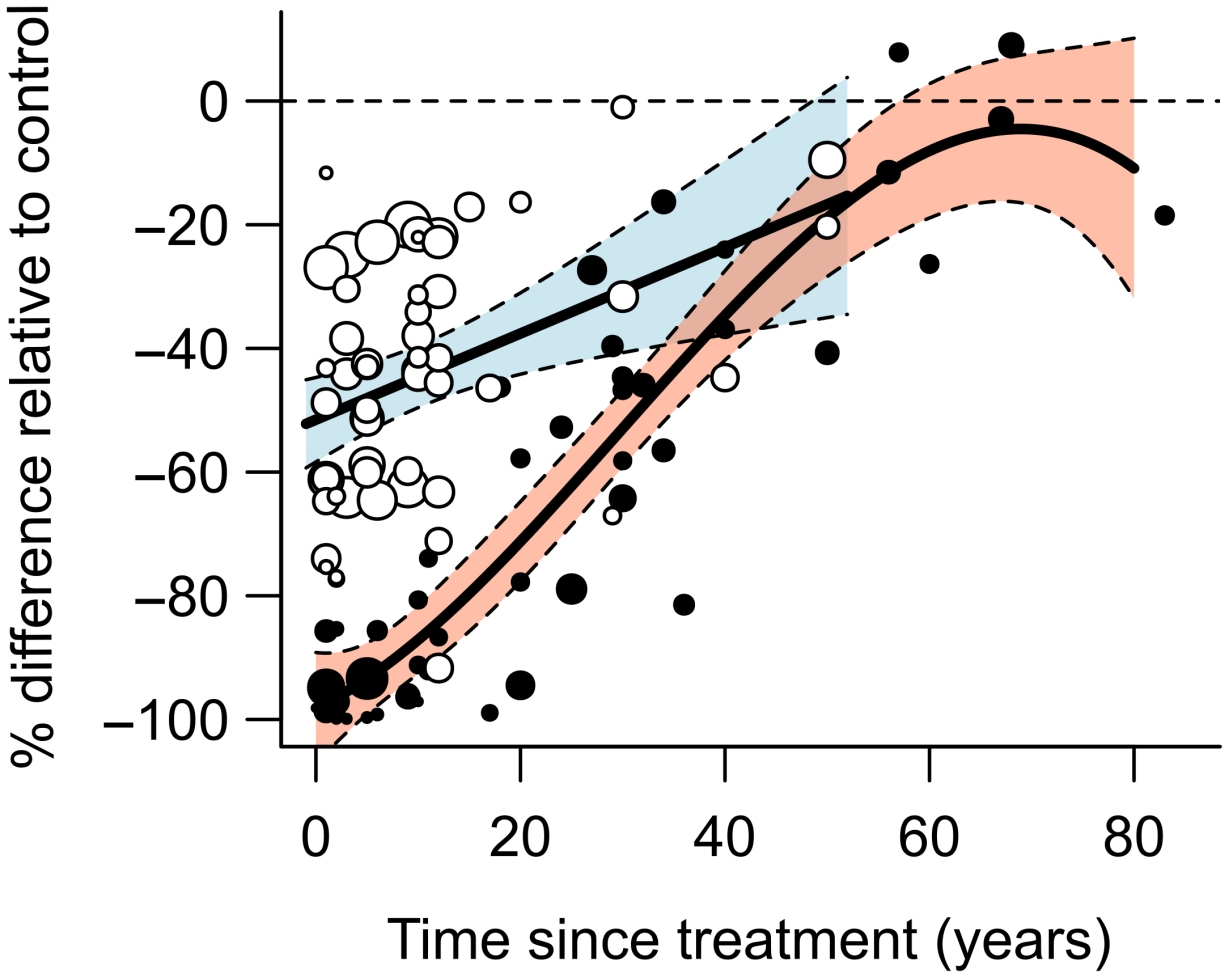
Effect of clearcutting (black circles, black curve in the red shaded area) and partial cuttings (white circle, black line in the blue shaded area) as function of time since treatment on the percentage of change of live tree C relative to control, analyzed by a weighted meta‐regression model. The effect over time was best fitted with a linear regression for the partial cuttings and with a cubic spline (fitted by polynomial regression) for the clearcutting. Each meta‐regression was weighted by the inverse of the variance of each effect size, which is visualized by the size of each point where larger points correspond with smaller variance. Colored areas represent 95% CIs for each harvest treatment.

To confirm the relationship between live tree C pool and time since treatment, we also conducted a weighted meta‐regression on a subset of observations that had the necessary information to do so (i.e., number of replicates and variance associated with each reported C mean of a treatment). The best‐fitting model confirmed a linear relationship between the percentage difference in the live tree C pool and time since treatment in partial cuttings. By contrast, a cubic spline relationship was observed in clearcutting, with both treatments converging after 30 years (Figure 5).

Understory vegetation exhibited a high increase in effect size in the first 10 years since treatment, reaching 500% and 125% for clearcutting and partial cuttings, respectively, before returning to reference levels (Figure 4). Coarse woody debris demonstrated a dynamic that was similar in both harvest treatments, starting with an increase in C values in the years following harvest (136% and 43% for clearcutting and partial cuttings, respectively), followed by a slow decrease until reaching negative values compared to reference stands after 20–30 years since treatment (averaging −68% for both treatments). After this decrease, C values slowly increased again to return to reference values.

## DISCUSSION

The objective of our study was to provide a better understanding of the different responses of forest C pools to partial cuttings and clearcutting harvesting practices through a meta‐analysis of studies from Northeastern American boreal and temperate forests. We anticipated clear distinctions between the two harvesting methods on the ecosystem C stock. Our results indicated a −30% difference in total C post‐harvesting, predominantly influenced by C alterations in the overstory. Nonetheless, we did not identify significant differences in total C between the cutting treatments. Our study aligns with previous meta‐analyses (Kalies et al., 2016) and supports the observation that C in the mineral horizons, which constitutes a significant fraction of the ecosystem, showed no variation with the harvest treatments. Although total C did not differ between partial cuttings and clearcutting, we observed significant differences in C dynamics between the two practices, which are discussed further below.

### Carbon dynamics following harvesting

The C pool values recorded in this meta‐analysis align with the expected values for natural forests in North America. According to the FAO (2020), the largest pool should be expected in the soil (49.8%, compared to 47.8% observed in total in the forest floor and mineral horizon pools in this meta‐analysis), followed by living biomass and dead wood. Following harvesting, almost all of these pools showed some variability. Still, only tree biomass was clearly affected by the cutting intensity, with clearcutting leading to lower total C pool values than partial cuttings.

### Live trees

Immediately after treatment, the change relative to the reference stand was greater in clearcutting, with values similar to those obtained in other studies (Zhou et al., 2013). It is widely acknowledged that post‐harvest biomass C initially accumulates slowly and then accelerates before plateauing, followed by a slowdown in accumulation (Seedre et al., 2011); this pattern was notably observed in boreal forests of Quebec (Senez‐Gagnon et al., 2018). In our study, this pattern was particularly pronounced in the clearcutting, with an expected period of 60 years to rebuild the C pool. Nash et al. (2024) also observed a rapid increase in C accumulation after clearcutting, reaching levels similar to those of reference stands by 60 years post‐treatment. However, C accumulation under partial cutting was slower than anticipated. Interestingly, the faster C accumulation in clearcutting compared to partial cuttings resulted in similar changes relative to the reference stand between the two treatments after 30–40 years. This outcome contrasts with some modeling studies that suggest higher (Schwenk et al., 2012) or similar (Puhlick et al., 2020) annual C accumulation in partial cuttings compared to clearcut stands. However, similar trends to our results were observed in simulated hardwood stands when comparing the merchantable basal area of partial cutting stands to those subjected to a more severe cutting (Bédard et al., 2014).

Empirical studies focusing on annual growth rates across different partial cutting intensities generally observe nuanced and non‐significant results. For instance, Sendak et al. (2003) found that annual increment tends to decrease with increasing harvest intensity, while others observed no differences (Erdmann & Oberg, 1973) or a trend toward higher annual increments (Bédard et al., 2018; Pothier, 1996). In a meta‐analysis focusing on partial harvesting, Zhou et al. (2013) did not find a significant recovery trend after low‐intensity cutting but observed an increased rate of C accumulation with higher cutting intensity. However, as in our study, the limited availability of data for partial cuttings beyond 20 years post‐harvest may have restricted the ability to estimate long‐term recovery trends and added variability to the results. Furthermore, differences in partial cutting practices, forest type, and stand structure can potentially influence the effects of partial cuttings (Ameray et al., 2021; Nunery & Keeton, 2010; Powers et al., 2011). For example, Moussaoui et al. (2020) found that stand structure and site characteristics determine the effectiveness of partial cuttings in boreal forests, with outcomes ranging from increased tree recruitment and growth to a loss of stand volume due to tree mortality. Site characteristics have also been identified as important predictors of C storage (Grant, 2004; Nunery & Keeton, 2010; Wang et al., 2003).

### Understory vegetation

The C dynamics of understory vegetation exhibited a greater change in clearcutting compared to partial cutting treatments. A similar magnitude of relative differences between partial cuttings and clearcutting was obtained by Ola et al. (2024). Understory vegetation is significantly influenced by disturbances (Hart & Chen, 2008); following a clearcut, the substantial change in light availability should result in a rapid increase in vegetation biomass (Seedre & Chen, 2010). Over time after cutting, forest succession leads to canopy closure, consequently causing a subsequent decrease in understory biomass vegetation (Fleming & Freedman, 1998). Since partial cuttings result in lower light availability due to a smaller canopy opening compared to clearcutting, it is expected to see less biomass accumulation in the understory. However, even though understory production can exceed the overstory production (O'connell et al., 2003), it represents only a small fraction of the total C biomass (Gilliam, 2007; Powers et al., 2011), thus carrying negligible weight in the comparison of both cutting methods relative to reference stands.

### Dead biomass

The principal factor determining changes in deadwood C pool was time since treatment. Deadwood C accumulation after cutting is known to be greatly influenced by the time since treatment and can follow different patterns depending on stand species composition (Seedre et al., 2011). Despite comparing different cutting methods in contrasting forest biomes, our results depicted a somewhat clear global “U”‐ or boomerang‐shaped debris C accumulation curve, considered the most common dynamic (Hély et al., 2000; Russell et al., 2015; Seedre et al., 2011; Senez‐Gagnon et al., 2018). The initial increase could be attributed to residues left after logging, while the subsequent decrease is due to their decomposition, as observed by Mattson et al. (1987) in temperate hardwood forests. The later increase may be due to intensified competition between trees, followed by the onset of senescence, which leads to tree mortality.

Differences in the amount of deadwood can contribute to the whole C ecosystem by increasing stand productivity in the long term through nutrient availability (Achat et al., 2015; Harmon et al., 1986; Ouimet et al., 2021), although negative relationships were also observed (Childs & Flint, 1990). In that regard, we expected different patterns of deadwood C dynamics between harvest treatments, as greater input following clearcutting is expected due to the intensity of harvest (Mund & Schulze, 2006). Interestingly, the pattern remained consistent between the two harvest treatments, though it may have been modulated by the harvest method used, that is, whole‐tree harvest (in which the tree is cut and forwarded to the roadside, where it is delimbed and topped) versus bole‐only (in which the tree is cut, delimbed, and topped at the stump), as well as the machines used for the harvesting operations. Both treatments resulted in a decrease in the C pool of snags primarily due to the removal of snags during harvesting. However, it is possible to limit this decline if retention directives are implemented.

Although not significant, it should be noted that a trend for higher deadwood C was observed in boreal forest stands compared to temperate forests. In boreal forests, the higher proportion of coniferous species leads to slower decomposition that may take more than 30 years (Hagemann et al., 2009; Moroni, 2006; Strukelj et al., 2015).

### Soil

In our study, organic C stored in the forest floor was primarily affected by forest biome irrespective of treatment, although we did observe a negative effect of clearcutting in temperate forest ecosystems. The recorded −21% difference in forest floor C pool relative to reference stands is consistent with findings from previous meta‐analyses (James et al., 2021; Nave et al., 2010), which reported up to 30% losses depending on soil type and dominant species. In their recent literature review, Mayer et al. (2020) suggest that reduced C stocks following clearcutting may be attributed to diminished litter input and/or increased decomposition compared to partial harvesting. However, the main driving factor appears to be related to site preparation and disturbance (Achat et al., 2015; James & Harrison, 2016) rather than cutting intensity per se. In our study, which was restricted to sites that received no soil preparation or fertilization, forest floor C in boreal forests appeared relatively stable despite harvesting. Forest floor serves as a major C pool in boreal forests, and in some stands, the organic C is characterized by stable forms that may limit the effect of harvesting or accelerate their recovery (Laganiere et al., 2013; Strukelj et al., 2015).

On the other hand, the mineral horizon C levels of the harvested stands were similar to those of the reference stands regardless of the harvesting practice, biome consideration, or sampling depth. This result is consistent with that of previous findings across different cutting practices and forest ecosystems (James et al., 2021; Mayer et al., 2020; Nave et al., 2010; Zhou et al., 2013). C pool in the mineral horizons can vary greatly between sites (Diochon et al., 2009) and is primarily determined by soil chemistry and physical characteristics (Nave et al., 2010). Soil alteration in this horizon is a slow process that occurs over centuries, so the cutting intensity may not exert sufficient pressure yet to affect this C pool. This emphasizes the importance of maintaining long‐term research sites in managed forests across a variety of soil types (Clarke et al., 2015). It is also important to note that the studies included in this meta‐analysis exhibited considerable variation in sampling depth, ranging from 7 to 150 cm. While the impact of sampling depth could not be assessed in this study due to insufficient data for each harvesting treatment, it remains a knowledge gap that needs further investigation, as also highlighted in other meta‐analyses and reviews (James et al., 2021; Mayer et al., 2020).

### Cutting cycle length

Forest ecosystems develop slowly, and research evaluating the effects of management practices thus requires decades to yield meaningful findings. Our study chose to compare C dynamics, as described by empirical data, following one cycle of harvest practices. This approach offered the benefit of assessing each cutting effect on the same basis, suggesting that potential aspects of partial cuttings over clearcutting on live C pools may depend on the cutting cycle length. Furthermore, the effects of cutting practices may yield different results when multiple cycles are considered, such as on soil or deadwood stocks. Some of the chronosequences available in the literature compared cutting treatments with different cycle lengths and time since treatment to provide a point‐in‐time assessment of the C budget following long‐term forest management. For example, on a 50‐year chronosequence with multiple cutting cycles, Puhlick et al. (2016) found that ecosystem C was still higher in natural, unmanaged stands, followed by the shelterwood method, selection cutting, and clearcutting. Similarly, Powers et al. (2011) found that long‐term management reduced total ecosystem C stocks, even after considering harvested wood products. Experimental sites in different conditions and with a variety of cutting methods and cycle lengths are crucial for enhancing our understanding of forest management's C dynamics over extended periods. However, these experiments are constrained by the initial choice of cutting cycle length, which may not align with the most optimal practices in modern forestry, given its evolving nature. As they are, such experiments work against long‐term commitments to fund and maintain study sites, making them rare despite their usefulness in meta‐analyses (Powers & Van Cleve, 1991).

### Implications and limitations

Proper forest management has the potential to contribute to increasing C stocks and sequestration and reducing C emissions (Perez‐Garcia et al., 2005). With increasing timber demands and a shift toward harvest practices such as partial cuttings, it becomes crucial to comprehensively understand their effects and implement them in a manner that ensures long‐term sustainability. The results of our meta‐analysis affirm that enhancing C storage in managed forest ecosystems will depend on allowing sufficient time for forests to recover (Harmon et al., 2009; Zhou et al., 2013). Our results showed that after 30 years, these stands surprisingly did not have C stocks comparable to those of unmanaged stands. However, forest stands managed by partial cuttings often follow a cutting cycle inferior to 30 years. This suggests that using partial cuttings instead of clearcutting to mitigate the effects of forest management on C pools may be more complex than previously thought. Further studies are needed to first identify suitable forest stands for partial cutting and afterward to evaluate the tree selection strategies (in terms of tree diameter, species, and health) to optimize forest productivity and C sequestration.

While we focused solely on C pools, other variables are also necessary to inform management policies, such as ecosystem C fluxes, fossil emissions from forest operations, transport and wood processing, and the fate of C stored in wood products during their life cycle (Moreau, Thiffault, & Beauregard, 2023; Moreau, Thiffault, Kurz, et al., 2023). Choosing partial cuttings may also entail expanding managed areas to sustain the same wood production (Lindenmayer et al., 2012), resulting in additional emissions, although this is not always the case (Giasson et al., 2023). The application of partial cuttings can also be driven by other objectives, such as managing species composition (Bédard et al., 2022; Sendak et al., 2003) and maintaining or diversifying stand structure (Bédard et al., 2014; Gauthier et al., 2015) to improve resistance and resilience to natural disturbances (Mohr et al., 2024). Nevertheless, partial cuttings are not the only solution; it is one of many tools available for managing forests depending on the relative importance of the desired objectives (Schwenk et al., 2012).

Our results need to be nuanced by the fact that “partial cutting” treatments used in this meta‐analysis encompassed contrasting methods regarding forest management objectives and intensities (Bose et al., 2013) applied under various site conditions. For example, we combined shelterwood treatments—intended to ensure stand renewal through one or more overwood removals, sometimes resulting in the final removal of the overwood or retaining a continuous cover—with light commercial thinning treatments, which aim to promote the growth of residuals trees by removing as little as 10% of the basal area. Ideally, each specific partial cutting method should have been analyzed independently. However, not enough studies or information about the practices were available for each treatment to standardize both the environmental conditions and the time since treatment. Pooling everything together certainly introduced some variability that may have hindered the discovery of certain inferences, representing a limitation of this meta‐analysis. On the other hand, despite this added variability, some differences were still detected, highlighting the significance of the analyzed factors. Similarly, the studies included in this meta‐analysis focused on commercial clearcutting. However, when clearcutting is combined with soil preparation and artificial regeneration, such as direct seeding or planting seedlings, it could be expected to lead to a faster recovery of C levels comparable to those of reference stands.

As reported by previous literature reviews and meta‐analyses (Mayer et al., 2020; Zhou et al., 2013), the number of studies reporting C values for soil mineral horizons was sparse and limited despite its importance. While of lesser importance, reports on understory vegetation were also limited. Much of the literature studying the harvest impact on understory vegetation focused solely on diversity changes, which may be attributed to the difficulty of estimating C biomass for this pool. Many studies in our database also lacked detailed information on the forestry practices used, proper referencing, or replication, greatly limiting both the precision and exploration of correlated variable effects (such as reference type used or site preparation) in this meta‐analysis. Finally, our inferences were significantly impeded by the limited availability of long‐term experimental designs and chronosequences. Most long‐term studies are centered mainly on undisturbed natural habitats (Powers & Van Cleve, 1991). This once again emphasizes their crucial need and importance in a variety of sites and treatments for being able to perform meta‐analyses on C sequestration and storage.

## CONFLICT OF INTEREST STATEMENT

The authors declare no conflicts of interest.

## Supporting information


Appendix S1.

Appendix S2.

## Data Availability

Data (Collin, 2025) are available on Zenodo at https://doi.org/10.5281/zenodo.15271337.
