# Supplementary material for: Effects of harvest treatments on forest carbon pools in eastern North America: A meta‐analysis
Source: Ecol Appl. 2025 May 26;35(3):e70050. doi: 10.1002/eap.70050 (PMC12104859; doi:10.1002/eap.70050)
Supplement: Supplementary file 1 — Appendix S1. [file EAP-35-e70050-s002.pdf]

APPENDIX S1

Alexandre Collin, Evelyne Thiffault, Stéphane Tremblay, Frédérik Doyon, Philippe Nolet.

Effects of harvest treatments on forest carbon pools in Eastern North America: A meta-analysis

Ecological Applications

Table S1: Publications included in this meta-analysis. C pool abbreviations: LT, Live trees; S, Snags; U, Understory vegetation; D, Coarse Woody Debris; FF, Forest floor; MH, Mineral horizons.

Reference	Location	Forest biome	Treatment	Cutting type	Time since harvest (years)	C Pool measured	*n*	Standard deviation	Used for total ecosystem C	Type of forest used as reference
Alban & Perala (1992)	MN, USA	Temperate	Clearcut	Stem only	0, 5, 7	LT, S, U, D, FF	1	no	no	Unmanaged
Angers et al. (2005)	QC, Canada	Temperate	Partial cut	Selection	12, 30	LT, S, D	6	yes	no	Old forest
				Diameter limit	12, 30	LT, S, D	6	yes	no	Old forest
Archambault et al. (1998)	QC, Canada	Boreal	Clearcut	Whole tree	20	LT	10	no	no	Unmanaged
Archambault et al. (2003)	QC, Canada	Temperate	Partial cut	Selection	50	LT	6	yes	no	Unmanaged
Audet-Giroux (2021)	QC, Canada	Boreal	Clearcut	Stem only	32	LT, S, U, D, FF, MH	41	yes	yes	Unmanaged
			Partial cut	Selection	10	LT, S, U, D, FF, MH	18	yes	yes	Unmanaged
Bédard & Majcen (2001)	QC, Canada	Temperate	Partial cut	Selection	10	LT	5	yes	no	Unmanaged
Borman & Likens (2012)	NH, USA	Temperate	Clearcut	-	1, 55	S, D	5	yes	no	Old forest
Bose et al. (2014)	QC, Canada	Boreal	Partial cut	Selection	12	LT, S	6	yes	no	Old forest
Brais et al. (2004)	QC, Canada	Boreal	Clearcut	Stem only	1	D	3	yes	no	Old forest
			Partial cut	Selection	1	D	6	yes	no	Old forest
Burgess et al. (2005)	ON, Canada	Temperate	Partial cut	Selection	1, 10, 20, 30	LT	3	yes	no	Old forest
Chen & Shrestha (2012)	ON, Canada	Boreal	Clearcut	-	2, 10, 29	FF	3	yes	no	Old forest
Covington (1981)	NH, USA	Temperate	Clearcut	-	3, 4, 7, 11, 18, 19, 22, 30, 35, 40, 44, 49, 57	FF	30	yes	no	Old forest
Diochon et al. (2009)	NS, Canada	Temperate	Clearcut	Stem only	1, 15, 45, 80	LT, FF, MH	1	no	no	Old forest
Federer (1984)	NH, USA	Temperate	Clearcut	-	2, 4, 10, 13, 24, 31, 34, 40, 44, 70	FF	5	yes	no	Unmanaged
Fortin et al. (2003)	QC, Canada	Temperate	Partial cut	Diameter limit	10	LT	2	yes	no	Unknown
Fraver et al. (2002)	ME, USA	Temperate	Partial cut	Gap harvesting	3	D	6	yes	no	Unmanaged
Goodburn & Lorimer (1998)	MI, USA	Temperate	Partial cut	Selection	12	LT, S, D	10	yes	no	Unmanaged

Gore & Patterson III (1986)	NH, USA	Temperate	Clearcut	-	1, 15, 50, 100	D	3	yes	no	Old forest
Gough et al. (2007)	MI, USA	Temperate	Clearcut	Stem only	6, 24, 50, 56	LT, U, D, FF, MH	5	yes	yes	Unmanaged
Hagemann et al. (2009)	NL, Canada	Boreal	Clearcut	-	1, 17, 36	LT, S, D, FF, MH	3	yes	yes	Unmanaged
Hendrickson (1988)	ON, Canada	Temperate	Clearcut	Whole tree	4	LT	1	no	no	Unmanaged
			Clearcut	Stem only	4	LT	1	no	no	Unmanaged
Hendrickson et al. (1989)	ON, Canada	Temperate	Clearcut	Whole tree	3	FF, MH	1	no	no	Unmanaged
			Clearcut	Stem only	3	FF, MH	1	no	no	Unmanaged
Howard et al. (2004)	SK, Canada	Boreal	Clearcut	Stem only	0, 5, 10, 29	LT, S, U, D, FF, MH	4	yes	yes	Unmanaged
Huntington & Ryan (1990)	NH, USA	Temperate	Clearcut	Whole tree	3	FF, MH	6	yes	no	Unmanaged
Johnson et al. (1991)	NH, USA	Temperate	Clearcut	Whole tree	3	FF	6	no	no	Unmanaged
Kishchuk et al. (2015)	AB, Canada	Boreal	Clearcut	-	10	FF, MH	3	yes	no	Unknown
Klockow et al. (2013)	MN, USA	Temperate	Clearcut	Whole tree	1	LT, S, U, D, FF, MH	4	yes	yes	Unmanaged
			Clearcut	Stem only	1	LT, S, U, D, FF, MH	4	yes	yes	Unmanaged
Lafleur et al. (2019)	QC, Canada	Temperate	Clearcut	-	12	LT	3	yes	no	Unknown
			Partial cut	Diameter limit	12	LT	3	yes	no	Unknown
			Partial cut	Gap harvesting	12	LT	3	yes	no	Unknown
Lee et al. (2002)	ON, Canada	Boreal	Clearcut	Whole tree	1, 3, 4, 5, 7	LT, S, U, D, FF	3	yes	no	Unmanaged
			Partial cut	Shelterwood	1, 3, 4, 5, 7	LT, S, U, D, FF	3	yes	no	Unmanaged
Majcen & Richard (1992)	QC, Canada	Temperate	Partial cut	Selection	5	LT	3	yes	no	Unmanaged
Majcen & Bédard (2000)	QC, Canada	Temperate	Partial cut	Selection	15	LT	3	yes	no	Unmanaged
Maleki et al. (2020)	QC, Canada	Temperate	Clearcut	-	1, 12	S	3	yes	no	Unknown
			Partial cut	Diameter limit	1, 12	S	3	yes	no	Unknown
			Partial cut	Gap harvesting	1, 12	S	3	yes	no	Unknown
Martin et al. (2005)	MB, Canada	Boreal	Clearcut	-	11, 18, 30	LT, U, D, FF, MH	4	yes	yes	Unknown
Matsuzaki et al. (2013)	BC, Canada	Temperate	Clearcut	-	1	LT, S, D, FF	4	yes	no	Old forest
			Partial cut	Selection	1	LT, S, D, FF	8	yes	no	Old forest
Mcgee et al. (1999)	NY, USA	Temperate	Partial cut	Selection	17	LT, S, D	6	yes	no	Old forest

McLaughlin & Phillips (2006)	ME, USA	Temperate	Clearcut	Whole tree	17	FF, MH	8	yes	no	Unmanaged
Moroni et al. (2010)	NL, Canada	Boreal	Clearcut	-	2, 34	LT, S, D, FF, MH	6	yes	yes	Unmanaged
Mroz et al. (1985)	MI, USA	Temperate	Clearcut	Whole tree	3	FF	3	yes	no	Unmanaged
Pamerleau-Couture et al. (2015)	QC, Canada	Boreal	Partial cut	Diameter limit	5, 9	LT, S	10	yes	no	Unmanaged
			Partial cut	Selection	10	LT, S	5	yes	no	Unmanaged
Payne et al. (2019)	ON, Canada	Boreal	Clearcut	-	2, 20, 25	LT, S, U, D, FF, MH	8	yes	yes	Old forest
Pennock & van Kessel (1997)	SK, Canada	Boreal	Clearcut	-	2, 13	FF	4	yes	no	Old forest
Powers et al. (2011)	WI, USA	Temperate	Partial cut	Diameter limit	12	LT, U, D, FF, MH	3	yes	yes	Old forest
			Partial cut	Shelterwood	29	LT, U, D, FF, MH	3	yes	yes	Old forest
			Partial cut	Selection	3	LT, U, D, FF, MH	9	yes	yes	Old forest
Prest et al. (2014)	NS, Canada	Temperate	Clearcut	Stem only	35	FF, MH	3	yes	no	Unmanaged
Prevost et al. (2010)	QC, Canada	Temperate	Partial cut	Selection	1, 5, 10	LT	12	yes	no	Unmanaged
Puhlick et al. (2016)	ME, USA	Temperate	Clearcut	Stem only	30	LT, U, D, FF, MH	4	yes	yes	Unmanaged
			Partial cut	Selection	5	LT, U, D, FF, MH	4	yes	yes	Unmanaged
			Partial cut	Shelterwood	40	LT, U, D, FF, MH	4	yes	yes	Unmanaged
Puhlick et al. (2022)	ME, USA	Temperate	Clearcut	-	2	LT, S, D	3	yes	no	Unmanaged
			Partial cut	Shelterwood	2	LT, S, D	3	yes	no	Unmanaged
			Partial cut	Selection	2	LT, S, D	3	yes	no	Unmanaged
			Partial cut	Diameter limit	2	LT, S, D	3	yes	no	Unmanaged
Roberge (1975)	QC, Canada	Temperate	Partial cut	Selection	1, 5, 10	LT	8	no	no	Unmanaged
Roskoski (1977)	NH, USA	Temperate	Clearcut	-	4, 8, 18, 40, 57	D	5	yes	no	Old forest
Rothstein & Spaulding (2010)	MI, USA	Temperate	Clearcut	Whole tree	5, 15	FF, MH	3	yes	no	Unmanaged
Roy et al. (2021)	QC, Canada	Temperate	Clearcut	-	5, 15, 30	D	27	yes	no	Old forest
			Partial cut	Selection	5, 15, 30	D	27	yes	no	Old forest
Seedre & Chen (2010)	ON, Canada	Boreal	Clearcut	-	1, 9, 27	LT, U	3	yes	no	Old forest
Senez-Gagnon et al. (2018)	QC, Canada	Boreal	Clearcut	Stem only	1, 10, 20, 30, 40, 67, 68	LT, S, U, D, FF, MH	4	yes	yes	Unmanaged
Shrestha & Chen (2010)	ON, Canada	Boreal	Clearcut	-	2, 10, 28	FF	3	yes	no	Old forest

St-Laurent et al. (2000)	QC, Canada	Temperate	Clearcut	Whole tree	7, 12, 22	FF, MH	10	yes	no	Old forest
Strong (1997)	WI, USA	Temperate	Partial cut	Diameter limit	40	LT, S, U	3	no	no	Old forest
			Partial cut	Selection	10	LT, S, U	9	no	no	Old forest
Strukelj et al. (2015)	QC, Canada	Boreal	Clearcut	Stem only	1, 3, 6, 9	LT, S, D, FF, MH	3	yes	yes	Old forest
			Partial cut	Selection	1, 3, 6, 9	LT, S, D, FF, MH	6	yes	yes	Old forest
Tattersall Smith et al. (2022)	ME, USA	Temperate	Clearcut	Whole tree	35	FF	4	no	no	Old forest
			Clearcut	Stem only	35	FF	4	no	no	Old forest
Taylor et al. (2007)	NS, Canada	Temperate	Clearcut	Stem only	35	FF	4	no	no	Old forest
Trettin et al. (2011)	MI, USA	Temperate	Clearcut	Whole tree	11	LT, U, D, FF, MH	3	yes	yes	Old forest
Tritton (1980)	NH, USA	Temperate	Clearcut	-	10, 20, 30, 40, 57, 60, 83	LT, S, D	31	yes	no	Old forest
Zha et al. (2009)	SK, Canada	Boreal	Clearcut	Stem only	2, 10, 29	S, D, FF, MH	3	yes	no	Old forest

References

Alban, D. H., & Perala, D. (1992). Carbon storage in Lake States aspen ecosystems. *Canadian Journal of Forest Research*, *22*(8), 1107-1110.

Angers, V. A., Messier, C., Beaudet, M., & Leduc, A. (2005). Comparing composition and structure in old-growth and harvested (selection and diameter-limit cuts) northern hardwood stands in Quebec. *Forest ecology and Management*, *217*(2-3), 275-293.

Archambault, L., Bégin, J., Delisle, C., & Fortin, M. (2003). Dynamique forestière après coupe partielle dans la Forêt expérimentale du Lac Édouard, Parc de la Mauricie, Québec. *The Forestry Chronicle*, *79*(3), 672-684.

Archambault, L., Morissette, J., & Bernier-Cardou, M. (1998). Forest succession over a 20-year period following clearcutting in balsam fir-yellow birch ecosystems of eastern Québec, Canada. *Forest ecology and Management*, *102*(1), 61-74.

Audet-Giroux, V. (2021). *La séquestration du carbone dans les écosystèmes de la forêt boréale selon les traitements sylvicoles* [Mémoire de Maîtrise, Université Laval].

Bédard, S., & Majcen, Z. (2001). Ten-year response of sugar maple–yellow birch–beech stands to selection cutting in Québec. *Northern Journal of Applied Forestry*, *18*(4), 119-126.

Bormann, F. H., & Likens, G. E. (2012). *Pattern and process in a forested ecosystem: disturbance, development and the steady state based on the Hubbard Brook ecosystem study*. Springer Science & Business Media.

Bose, A. K., Brais, S., & Harvey, B. D. (2014). Trembling aspen (Populus tremuloides Michx.) volume growth in the boreal mixedwood: Effect of partial harvesting, tree social status, and neighborhood competition. *Forest ecology and Management*, *327*, 209-220.

Brais, S., Harvey, B., Bergeron, Y., Messier, C., Greene, D., Belleau, A., & Paré, D. (2004). Testing forest ecosystem management in boreal mixedwoods of northwestern Quebec: initial response of aspen stands to different levels of harvesting. *Canadian Journal of Forest Research*, *34*(2), 431-446.

Burgess, D., Robinson, C., & Wetzel, S. (2005). Eastern white pine response to release 30 years after partial harvesting in pine mixedwood forests. *Forest ecology and Management*, *209*(1-2), 117-129.

Chen, H. Y., & Shrestha, B. M. (2012). Stand age, fire and clearcutting affect soil organic carbon and aggregation of mineral soils in boreal forests. *Soil Biology and Biochemistry*, *50*, 149-157.

Covington, W. W. (1981). Changes in forest floor organic matter and nutrient content following clear cutting in northern hardwoods. *Ecology*, *62*(1), 41-48.

Diochon, A., Kellman, L., & Beltrami, H. (2009). Looking deeper: An investigation of soil carbon losses following harvesting from a managed northeastern red spruce (Picea rubens Sarg.) forest chronosequence. *Forest ecology and Management*, *257*(2), 413-420.

Federer, C. A. (1984). Organic matter and nitrogen content of the forest floor in even-aged northern hardwoods. *Canadian Journal of Forest Research*, *14*(6), 763-767.

Fortin, M., Bégin, J., & Bélanger, L. (2003). Les coupes partielles: une alternative à la coupe à blanc dans les peuplements mixtes de sapin baumier et d'épinette rouge en termes de rendement. *The Forestry Chronicle*, *79*(5), 948-956.

Fraver, S., Wagner, R. G., & Day, M. (2002). Dynamics of coarse woody debris following gap harvesting in the Acadian forest of central Maine, USA. *Canadian Journal of Forest Research*, *32*(12), 2094-2105.

Goodburn, J. M., & Lorimer, C. G. (1998). Cavity trees and coarse woody debris in old-growth and managed northern hardwood forests in Wisconsin and Michigan. *Canadian Journal of Forest Research*, *28*(3), 427-438.

Gore, J. A., & Patterson III, W. A. (1986). Mass of downed wood in northern hardwood forests in New Hampshire: potential effects of forest management. *Canadian Journal of Forest Research*, *16*(2), 335-339.

Gough, C. M., Vogel, C. S., Harrold, K. H., George, K., & Curtis, P. S. (2007). The legacy of harvest and fire on ecosystem carbon storage in a north temperate forest. *Global change biology*, *13*(9), 1935-1949.

Hagemann, U., Moroni, M. T., & Makeschin, F. (2009). Deadwood abundance in Labrador high-boreal black spruce forests. *Canadian Journal of Forest Research*, *39*(1), 131-142.

Hendrickson, O. (1988). Biomass and nutrients in regenerating woody vegetation following whole-tree and conventional harvest in a northern mixed forest. *Canadian Journal of Forest Research*, *18*(11), 1427-1436.

Hendrickson, O., Chatarpaul, L., & Burgess, D. (1989). Nutrient cycling following whole-tree and conventional harvest in northern mixed forest. *Canadian Journal of Forest Research*, *19*(6), 725-735.

Howard, E. A., Gower, S. T., Foley, J. A., & Kucharik, C. J. (2004). Effects of logging on carbon dynamics of a jack pine forest in Saskatchewan, Canada. *Global change biology*, *10*(8), 1267-1284.

Huntington, T., & Ryan, D. (1990). Whole-tree-harvesting effects on soil nitrogen and carbon. *Forest ecology and Management*, *31*(4), 193-204.

Johnson, C. E., Johnson, A. H., Huntington, T. G., & Siccama, T. G. (1991). Whole-tree clear-cutting effects on soil horizons and organic-matter pools. *Soil Science Society of America Journal*, *55*(2), 497-502.

Kishchuk, B. E., Thiffault, E., Lorente, M., Quideau, S., Keddy, T., & Sidders, D. (2015). Decadal soil and stand response to fire, harvest, and salvage-logging disturbances in the western boreal mixedwood forest of Alberta, Canada. *Canadian Journal of Forest Research*, *45*(2), 141-152. https://doi.org/10.1139/cjfr-2014-0148

Klockow, P. A., D'Amato, A. W., & Bradford, J. B. (2013). Impacts of post-harvest slash and live-tree retention on biomass and nutrient stocks in Populus tremuloides Michx.-dominated forests, northern Minnesota, USA. *Forest ecology and Management*, *291*, 278-288.

Lafleur, B., Harvey, B. D., & Mazerolle, M. J. (2019). Partial cutting in mixedwood stands: Effects of treatment configuration and intensity on stand structure, regeneration, and tree mortality. *Journal of Sustainable Forestry*, *38*(3), 275-291.

Lee, J., Morrison, I. K., Leblanc, J.-D., Dumas, M. T., & Cameron, D. A. (2002). Carbon sequestration in trees and regrowth vegetation as affected by clearcut and partial cut harvesting in a second-growth boreal mixedwood. *Forest ecology and Management*, *169*(1-2), 83-101.

Majcen, Z., & Bédard, S. (2000). *Accroissement après 15 ans dans une érablière à la suite de coupes de jardinage de diverses intensités*. Direction de la recherche forestière, Forêt Québec.

Majcen, Z., & Richard, Y. (1992). Résultats après 5 ans d'un essai de coupe de jardinage dans une érablière. *Canadian Journal of Forest Research*, *22*(11), 1623-1629.

Maleki, K., Lafleur, B., Harvey, B. D., Mazerolle, M. J., & Fenton, N. J. (2020). Changes in Deadwood and Understory Vegetation 12 Years after Partial and Clearcut Harvesting in Mixedwood Stands of Western Quebec, Canada. *Forest Science*, *66*(3), 337-350.

Martin, J. L., Gower, S. T., Plaut, J., & Holmes, B. (2005). Carbon pools in a boreal mixedwood logging chronosequence. *Global change biology*, *11*(11), 1883-1894.

Matsuzaki, E., Sanborn, P., Fredeen, A. L., Shaw, C. H., & Hawkins, C. (2013). Carbon stocks in managed and unmanaged old-growth western redcedar and western hemlock stands of Canada's inland temperate rainforests. *Forest ecology and Management*, *297*, 108-119.

McGee, G. G., Leopold, D. J., & Nyland, R. D. (1999). Structural characteristics of old-growth, maturing, and partially cut northern hardwood forests. *Ecological Applications*, *9*(4), 1316-1329.

McLaughlin, J. W., & Phillips, S. A. (2006). Soil carbon, nitrogen, and base cation cycling 17 years after whole-tree harvesting in a low-elevation red spruce (Picea rubens)-balsam fir (Abies balsamea) forested watershed in central Maine, USA. *Forest ecology and Management*, *222*(1-3), 234-253.

Moroni, M. T., Shaw, C. H., & Otahal, P. (2010). Forest carbon stocks in Newfoundland boreal forests of harvest and natural disturbance origin I: field study. *Canadian Journal of Forest Research*, *40*(11), 2135-2145. https://doi.org/10.1139/x10-154

Mroz, G. D., Jurgensen, M. F., & Frederick, D. J. (1985). Soil Nutrient Changes Following Whole Tree Harvesting on Three Northern Hardwood Sites. *Soil Science Society of America Journal*, *49*(6), 1552-1557. https://doi.org/10.2136/sssaj1985.03615995004900060044x

Pamerleau-Couture, É., Krause, C., Pothier, D., & Weiskittel, A. (2015). Effect of three partial cutting practices on stand structure and growth of residual black spruce trees in north-eastern Quebec. *Forestry: An International Journal of Forest Research*, *88*(4), 471-483. https://doi.org/10.1093/forestry/cpv017

Payne, N. J., Allan Cameron, D., Leblanc, J.-D., & Morrison, I. K. (2019). Carbon storage and net primary productivity in Canadian boreal mixedwood stands. *Journal of Forestry Research*, *30*(5), 1667-1678. https://doi.org/10.1007/s11676-019-00886-0

Pennock, D. J., & van Kessel, C. (1997). Clear-cut forest harvest impacts on soil quality indicators in the mixedwood forest of Saskatchewan, Canada. *Geoderma*, *75*(1), 13-32. https://doi.org/10.1016/S0016-7061(96)00075-4

Powers, M., Kolka, R., Palik, B., McDonald, R., & Jurgensen, M. (2011). Long-term management impacts on carbon storage in Lake States forests. *Forest ecology and Management*, *262*(3), 424-431. https://doi.org/10.1016/j.foreco.2011.04.008

Prest, D., Kellman, L., & Lavigne, M. B. (2014). Mineral soil carbon and nitrogen still low three decades following clearcut harvesting in a typical Acadian Forest stand. *Geoderma*, *214-215*, 62-69. https://doi.org/10.1016/j.geoderma.2013.10.002

Prévost, M., Dumais, D., & Pothier, D. (2010). Growth and mortality following partial cutting in a trembling aspen – conifer stand: results after 10 years. *Canadian Journal of Forest Research*, *40*(5), 894-903. https://doi.org/10.1139/x10-036

Puhlick, J. J., Weiskittel, A. R., Fernandez, I. J., Fraver, S., Kenefic, L. S., Seymour, R. S., Kolka, R. K., Rustad, L. E., & Brissette, J. C. (2016). Long-term influence of alternative forest management treatments on total ecosystem and wood product carbon storage. *Canadian Journal of Forest Research*, *46*(11), 1404-1412. https://doi.org/10.1139/cjfr-2016-0193

Puhlick, J. J., Weiskittel, A. R., Fernandez, I. J., Solarik, K. A., & Sleep, D. J. H. (2022). Evaluation of projected carbon accumulation after implementing different forest management treatments in mixed-species stands in northern Maine. *Carbon Management*, *13*(1), 190-204. https://doi.org/10.1080/17583004.2022.2063761

Roberge, M. R. (1975). Effect of Thinning on the Production of High-quality Wood in a Quebec Northern Hardwood Stand. *Canadian Journal of Forest Research*, *5*(1), 139-145. https://doi.org/10.1139/x75-019

Roskoski, J. P. (1977). *NITROGEN FIXATION IN NORTHERN HARDWOOD FORESTS* (Publication Number 7728160) [Ph.D., Yale University]. ProQuest Dissertations & Theses Global. United States - Connecticut.

Rothstein, D. E., & Spaulding, S. E. (2010). Replacement of wildfire by whole-tree harvesting in jack pine forests: Effects on soil fertility and tree nutrition. *Forest ecology and Management*, *260*(7), 1164-1174. https://doi.org/10.1016/j.foreco.2010.07.007

Roy, M.-È., Surget-Groba, Y., Delagrange, S., & Rivest, D. (2021). Legacies of forest harvesting on soil properties along a chronosequence in a hardwood temperate forest. *Forest ecology and Management*, *496*, 119437. https://doi.org/10.1016/j.foreco.2021.119437

Seedre, M., & Chen, H. Y. H. (2010). Carbon dynamics of aboveground live vegetation of boreal mixedwoods after wildfire and clear-cutting. *Canadian Journal of Forest Research*, *40*(9), 1862-1869. https://doi.org/10.1139/x10-120

Senez-Gagnon, F., Thiffault, E., Paré, D., Achim, A., & Bergeron, Y. (2018). Dynamics of detrital carbon pools following harvesting of a humid eastern Canadian balsam fir boreal forest. *Forest ecology and Management*, *430*, 33-42. https://doi.org/10.1016/j.foreco.2018.07.044

Shrestha, B. M., & Chen, H. Y. H. (2010). Effects of stand age, wildfire and clearcut harvesting on forest floor in boreal mixedwood forests. *Plant and Soil*, *336*(1), 267-277. https://doi.org/10.1007/s11104-010-0475-2

St-Laurent, S., Ouimet, R., Tremblay, S., & Archambault, L. (2000). Évolution des stocks de carbone organique dans le solaprès coupe dans la sapinière à bouleau jaune de l'est du Québec. *Canadian Journal of Soil Science*, *80*(3), 507-514. https://doi.org/10.4141/s99-083

Strong, T. F. (1997). *Harvesting intensity influences the carbon distribution in a northern hardwood ecosystem* (Vol. 329). US Department of Agriculture, Forest Service, North Central Forest Experiment Station. http://dx.doi.org/10.2737/NC-RP-329

Strukelj, M., Brais, S., & Paré, D. (2015). Nine-year changes in carbon dynamics following different intensities of harvesting in boreal aspen stands. *European Journal of Forest Research*, *134*(5), 737-754. https://doi.org/10.1007/s10342-015-0880-4

Tattersall Smith, C., Briggs, R. D., Stupak, I., Preece, C., Rezai-Stevens, A., Barusco, B., Roth, B. E., Fernandez, I. J., & Simpson, M. J. (2022). Effects of whole-tree and stem-only clearcutting on forest floor and soil carbon and nutrients in a balsam fir (Abies balsamea (L.) Mill.) and red spruce (Picea rubens Sarg.) dominated ecosystem. *Forest ecology and Management*, *519*, 120325. https://doi.org/10.1016/j.foreco.2022.120325

Taylor, A. R., Wang, J. R., & Chen, H. Y. H. (2007). Carbon storage in a chronosequence of red spruce (Picea rubens) forests in central Nova Scotia, Canada. *Canadian Journal of Forest Research*, *37*(11), 2260-2269. https://doi.org/10.1139/x07-080

Trettin, C. C., Jurgensen, M. F., Gale, M. R., & McLaughlin, J. W. (2011). Recovery of carbon and nutrient pools in a northern forested wetland 11years after harvesting and site preparation. *Forest ecology and Management*, *262*(9), 1826-1833. https://doi.org/10.1016/j.foreco.2011.07.031

Tritton, L. M. (1980). *DEAD WOOD IN THE NORTHERN HARDWOOD FOREST ECOSYSTEM* (Publication Number 8025315) [Ph.D., Yale University]. ProQuest Dissertations & Theses Global. United States - Connecticut.

Zha, T., Barr, A. G., Black, T. A., McCaughey, J. H., Bhatti, J., Hawthorne, I., Krishnan, P., Kidston, J., Saigusa, N., Shashkov, A., & Nesic, Z. (2009). Carbon sequestration in boreal jack pine stands

following harvesting. *Global change biology*, *15*(6), 1475-1487. https://doi.org/10.1111/j.1365-2486.2008.01817.x
