# Supplementary material for: Effects of harvest treatments on forest carbon pools in eastern North America: A meta‐analysis
Source: Ecol Appl. 2025 May 26;35(3):e70050. doi: 10.1002/eap.70050 (PMC12104859; doi:10.1002/eap.70050)
Supplement: Supplementary file 2 — Appendix S2. [file EAP-35-e70050-s001.pdf]

## APPENDIX S2

Alexandre Collin, Evelyne Thiffault, Stéphane Tremblay, Frédérik Doyon, Philippe Nolet.

# Effects of harvest treatments on forest carbon pools in Eastern North America: A meta-analysis

*Ecological Applications*

**Table S1:** Number of effect size values reported by biome, cutting treatment and reference type.

| **Biome** | **Cutting treatment** | **Reference type** | ***n*** |
|---|---|---|---|
| Boreal | Clearcutting | Old forest | 125 |
| | | Unmanaged | 66 |
| | Partial cutting | Old forest | 15 |
| | | Unmanaged | 48 |
| Temperate | Clearcutting | Old forest | 120 |
| | | Unmanaged | 79 |
| | Partial cutting | Old forest | 60 |
| | | Unmanaged | 66 |

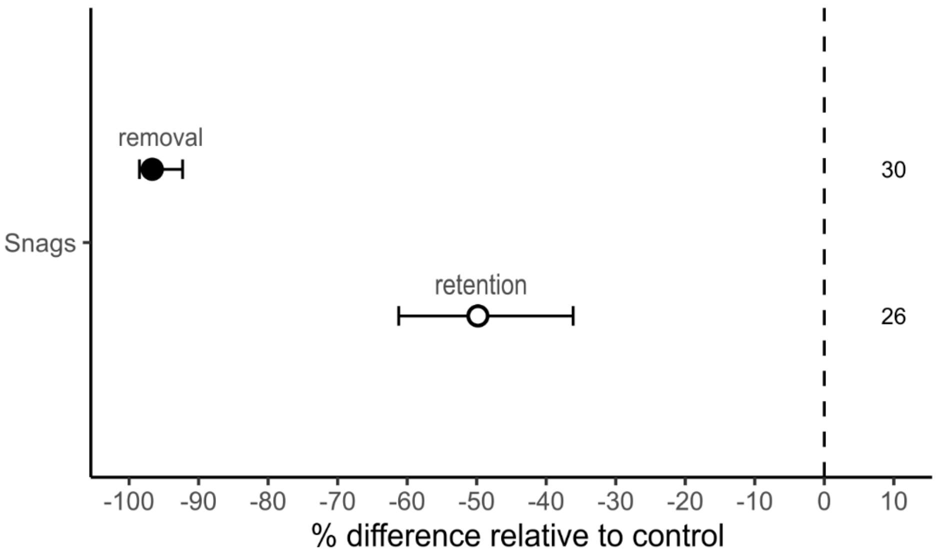


**Figure S1:** Effect of retention or removal of snag on the effect size for the subset of clearcutting where this information was available. Each estimate is shown with a bootstrapped 95% confidence interval. Each interval that does not overlap with the dotted vertical line indicates a statistically significant difference from the reference stand (i.e., old or unmanaged stand).

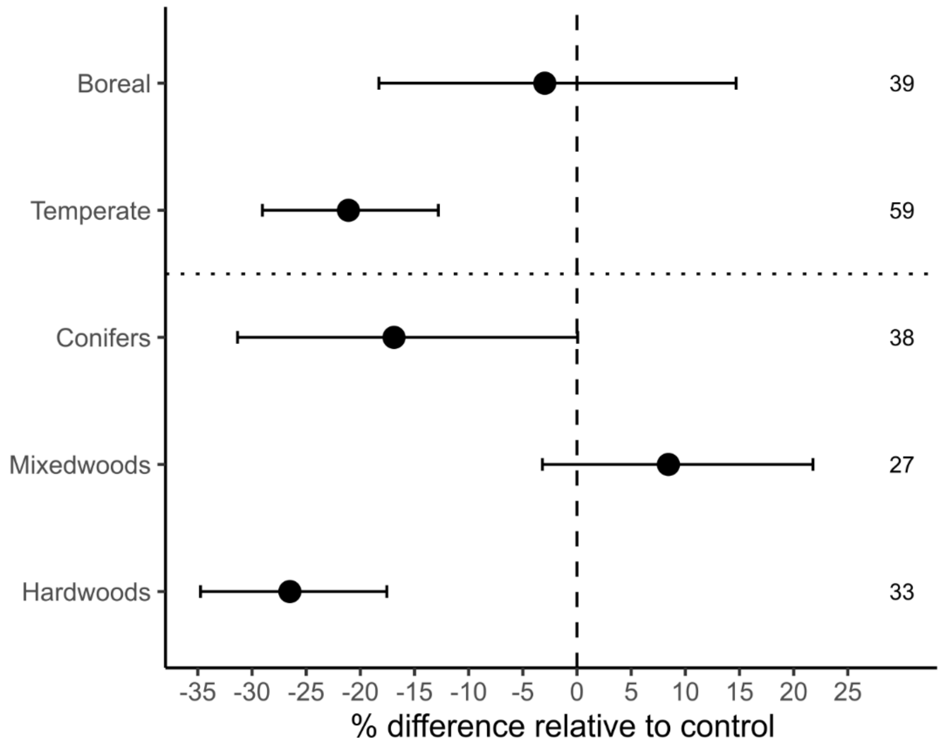


**Figure S2:** Effect of biome and forest type on the percentage difference in forest floor C relative to the reference stands. Each estimate is shown with a bootstrapped 95% confidence interval. Each interval that does not overlap with the dotted vertical line indicates a statistically significant difference from the reference stand (i.e., old or unmanaged stand). The number of observations from literature included in each estimate is listed on the right. Time since harvest was included as a random effect in the model.

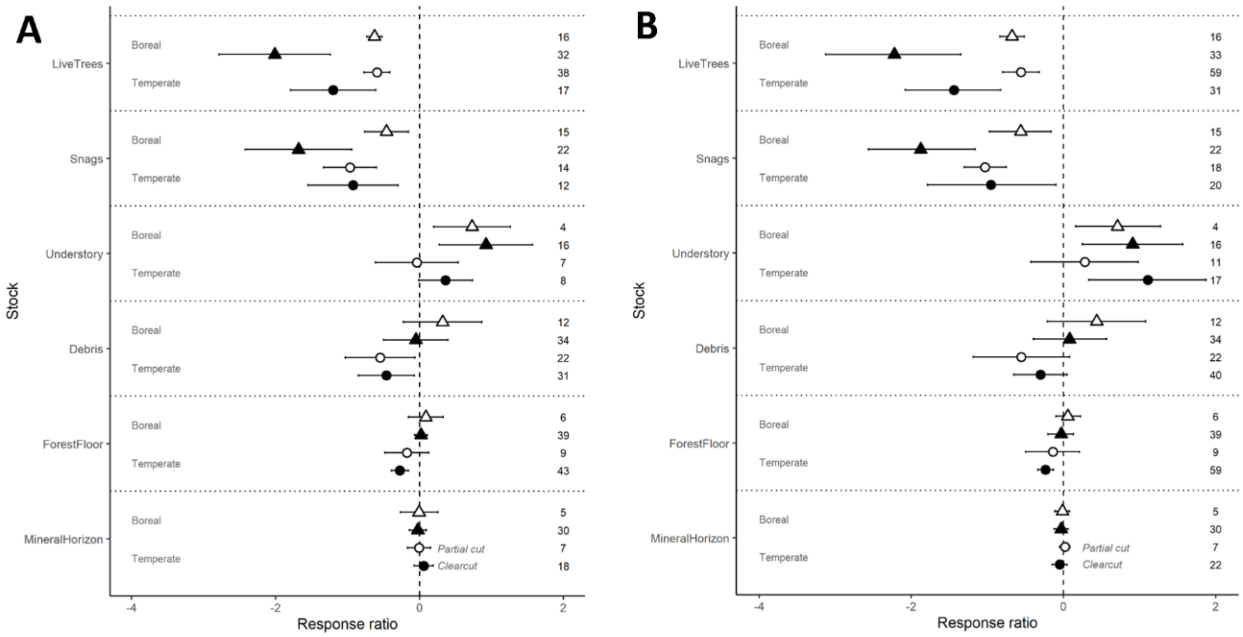


**Figure S3:** Comparison of weighted (A) and unweighted mixed effect (B) meta-analysis models on the C pool effect size following clearcutting (black symbols) or partial cuttings (white symbols) in boreal (triangles) or temperate (points) forests. Each estimate is shown with a bootstrapped 95% confidence interval. Each interval that does not overlap with the dotted vertical line indicates a statistically significant difference from the reference stand (i.e., old or unmanaged stand). The number of observations from literature included in each estimate is listed on the right. Time since treatment was included as a random effect in the model.
